# Organizational readiness for implementation: a qualitative assessment to explain survey responses

**DOI:** 10.1186/s12913-024-12149-8

**Published:** 2025-01-07

**Authors:** Maria McClam, Lauren Workman, Timothy J. Walker, Emanuelle M. Dias, Derek W. Craig, Joe R. Padilla, Andrea E. Lamont, Abraham Wandersman, Maria E. Fernandez

**Affiliations:** 1 Center for Applied Research and Evaluation, Arnold School of Public Health, University of South Carolina, Columbia, SC USA; 2 Department of Health Services, Policy, and Management, Arnold School of Public Health, University of South Carolina, Columbia, SC USA; 3 Center for Health Promotion and Prevention Research, School of Public Health, UT Health Houston, Houston, TX USA; 4 Wandersman Center, Columbia, SC USA

**Keywords:** Organizational readiness, Qualitative methods, Measure development, Evidence-based interventions, Cancer prevention, Social desirability, Implementation

## Abstract

**Background:**

One factor considered essential to successful implementation is organizational readiness. The purpose of this study was to explore ways to improve the measurement of organizational readiness, and in particular to refine a preliminary measure based on the Readiness = Motivation x innovation Specific Capacity x General Capacity (R = MC2) heuristic. We assessed the experiences of staff in Federally Qualified Health Centers (FQHC) implementing evidence-based interventions (EBIs) designed to increase colorectal cancer screening (CRCS) who previously completed the survey and aimed to understand their perspectives on why our data were positively skewed.

**Methods:**

We conducted a series of qualitative interviews with FQHC employees who had previously completed the readiness survey and/or been involved with the distribution of the readiness survey. Interviews were conducted via Zoom, recorded, transcribed. Data was analyzed using an inductive approach to identify key emergent themes.

**Results:**

Analyses revealed numerous contributors to high organizational readiness assessment scores including concerns about confidentiality, social desirability bias, knowledge of respondents about the survey content, and the survey format. Specific to the survey format, we found that survey length and response scaling likely impacted scores and the overall survey experience. Lastly, some participants shared that the tendency for high scores may reflect actual perceptions because FQHC staff are passionate, work well in teams, and typically have already worked hard to improve CRCS rates through evidence-based interventions.

**Conclusion:**

Study findings reinforce the importance of collaborative and community-engaged survey design and the need to address the common challenges dissemination and implementation surveys may be vulnerable to. Lessons learned can be applied to other measurement work and surveys conducted across public health research. The findings will inform continued organizational readiness measure development and have implications for measurement of other factors influencing implementation.

**Supplementary Information:**

The online version contains supplementary material available at 10.1186/s12913-024-12149-8.

## Background and purpose

Organizational readiness is a key factor in the success of innovations that organizations implement [1, 2]. Organizational readiness is defined as the ability and willingness of an organization to implement an innovation (i.e., practice change, intervention, policy that is new to the organization). Readiness has emerged as a key construct for successful implementation in multiple implementation science frameworks [2–6], including the R = MC^2^ framework [7]. The R = MC^2^ framework represents a synthesis and translation of the empirical literature on facilitators and barriers of implementation success and conceptualizes readiness as a combination of three overarching constructs (Readiness = Motivation x Innovation-Specific Capacity x General Capacity) [3, 8, 9], each of which is made up of multiple constructs known to be associated with implementation success. Although the importance of readiness is well-accepted in scientific communities, many empirical questions remain related to interconnections between constructs (e.g., structural relations) and how they relate to implementation outcomes and clinical success. The lack of psychometrically-strong instruments to measure readiness (and each respective subcomponent) hinders empirical progress in understanding readiness and, consequentially, the implementation process. Validated measures (developed with both internal and external validity in mind) of each readiness subcomponent are a necessary step to advance the science and practice of readiness for implementation.

Self-report surveys are a commonly used method to collect data from individuals and organizations. Surveys are useful because they allow for large amounts of data to be collected relatively quickly and can be administered in-person, over the phone, or online. Unfortunately, self-report surveys are susceptible to multiple types of bias and limitations that hinder the validity and reliability of research findings [10]. Issues such as survey length, confidentiality, and complexity can play a role in accuracy of response. There is a balance between comprehensive data collection and using a measurement tool that is pragmatic, or one that has relevance to stakeholders and is feasible to use in real-world settings [11]. Accuracy can be affected by many external factors, which need to be handled methodologically rather than statistically at times. For example, simple considerations such as familiarity with the innovation or where the organization is at in the process of implementing something new can play a big role on response patterns. Similarly, survey characteristics such as length and mental load play a role. Employees and staff may not have enough time in the workday to complete a long survey or may not prioritize responses because of being instructed by a supervisor to take the survey (versus completing it out of individual desire). In organizational research, issues of maintaining confidentiality and ensuring confidence that responses will not have negative repercussions on job performance evaluations are also often a concern. Social desirability bias, which occurs when participants overreport socially desirable attitudes and behaviors and underreport those deemed undesirable [7], is an important concern when conducting implementation research because employees/staff may not want to disclose weaknesses within their organization or want to appear positive in the eyes of supervisors. Biased results can lead to inaccurate conclusions that negatively affect future research, evaluations, and ultimately decisions related to the care provided to patients [12, 13]. Social desirability bias and other forms of error can be reduced by gathering feedback from a user’s perspective throughout the development process to inform modifications that improve the quality and pragmatism of the survey.

Federally qualified health centers (FQHCs) provide comprehensive healthcare services to underserved populations and communities throughout the United States [14]. FQHCs serve approximately 30 million patients annually and are therefore well-positioned to provide under and uninsured individuals with access to services needed to reduce many health disparities [15]. For instance, colorectal cancer screening (CRCS) uptake is significantly lower among patients who utilize FQHCs compared to the national average [16, 17]. Despite the availability of multiple evidence-based interventions (EBIs) designed to increase CRCS (e.g. patient reminders, provider reminders, provider assessment and feedback, and reducing structural barriers) [18], FQHCs face numerous challenges to implementing these interventions effectively [19–21]. Improving our understanding of what factors (like organizational readiness) influence the implementation of CRCS EBIs, and how, is a critical step toward increasing CRCS rates and reducing colorectal cancer-related deaths.

The goal of this study was to obtain information from survey respondents to inform the refinement of a new measure of organizational readiness. The measure tested in this study derived from an earlier version of a measure of R = MC^2^, which showed adequate preliminary psychometric properties but was originally developed for formative evaluation purposes and needed adaptations for inferential use [22]. This study is part of a broader study of measure development, representing the process of obtaining user input to optimize response accuracy and external validity. Specifically, we assessed the experiences of FQHC staff who completed a measure of organizational readiness, and aimed to understand their perspectives on why data were positively skewed. We also explored how social desirability bias may have contributed to the response profiles of readiness to implement CRCS EBIs in the FQHC setting. We sought to understand the extent to which the high scores (positively skewed) were accurate representations of the constructs being measured or whether it was reflective of some form of bias, survey feature (e.g., length, confidentiality, complexity), or clinic characteristics (e.g., the stage of implementation of the CRCS program in your clinic).

## Methods

We conducted a series of qualitative interviews (n = 16) with FQHC employees who had previously completed the readiness survey and/or been involved with the distribution of the readiness survey among FQHC staff. We adopted a phenomenological lens, a qualitative research approach focused on people’s experience rather than what meaning they assign to it later, to assess the experiences of the respondents while taking the survey in a healthcare environment [23, 24]. Research team members developed the interview guide based on expertise in measurement development and qualitative methods— no pre-determined framework was used. It is worth noting that as part of this larger study, our research team previously used findings from a series of cognitive interviews with FQHC staff to adapt the readiness survey used for survey data collection [25]. The readiness survey consisted of 167 readiness-specific questions across three main components. Subsequently, this series of interviews were conducted after survey data collection to understand survey experience, contextualize survey data, and further adapt the readiness survey for the validation phase.

### Participant recruitment

We invited employees from FQHCs who had participated in prior data collection with the readiness survey to participate in our study. The developmental phase consisted of data across 57 FQHC clinics located in New York, South Carolina, and Texas. Because there was a significant amount of time between when developmental sample data collection occurred (August, 2021 to April, 2022) and when these interviews were conducted (August, 2022 to January, 2023), three participants struggled to recall taking the survey but responded to interview questions by reflecting on surveys generally. We used a sampling framework informed by purposeful and maximum variation approaches to recruit clinic employees representing a range of different job types (e.g., clinic managers, quality improvement staff, nurses) [26, 27]. We emailed electronic and mailed paper versions of interview recruitment flyers to FQHC practice managers we worked with during the developmental phase and asked them to distribute the flyer to their staff who took the readiness survey during the developmental phase. A QR code was placed on the flyer for staff to sign up for an interview. Our research team originally offered interview participants a $30 gift card honorarium as a thank you for their time, but after a lack of interest we increased our gift card to $50. All data collected was kept confidential and no identifying information about FQHC participants was shared.

### Data collection

We developed a semi-structured interview guide designed to understand the survey experience. Our research team members developed the interview guide based on expertise in measurement development and qualitative methods. We did not use a pre-determined framework. Three research team members conducted interviews during August, 2022 to January, 2023 over a virtual meeting platform (Zoom) at a time convenient for each participant. Sample questions included general feedback such as, “*What was your general impression of the survey?*” and “*How clear was the information you received, including the survey purpose and directions?*” Additionally, we also inquired about how and why questions were answered in a way that favored a positive response. We prompted this by showing participants a visual of the survey data and high scores, then asked participants additional questions including “*Why do you think the ratings look the way they do?*”, “*We’ve wondered if the way we sent out the survey impacted the responses we got back. In a lot of instances, we worked with clinic managers to send out the survey to clinic staff. How, if it all, might this process have impacted the way people answered the survey?*”, and “*How might your responses to the survey have been impacted by where your clinics was in the process of implementing the CRCS program you were surveyed on?*”; the full interview guide is available as a supplementary file. Interviews lasted between 30 and 60 min and were audio recorded and transcribed verbatim for analysis.

### Data analysis

We coded all interview transcripts using a qualitative analysis software, Dedoose [28]. We used an inductive approach to analyze data wherein we identified emergent themes. We coded interview transcripts using the constant comparison technique, which calls for repeated review of text until meaningful categories emerge [29]. Two research team members independently coded a set of three transcripts and then met to compare interpretations of initial themes and emergent codes. After resolving differences, we developed a list of themes to focus the analysis. Then, we completed a more detailed analysis with another analysis of all transcripts. Data were reviewed until saturation [30] was reached (no new themes or ideas emerge from the data) and recurring themes were identified. Our analysis team used principles of researcher reflexivity [31] during data collection analysis, wherein we shared reflections, addressed interpretive inconsistencies, and regularly discussed different points of view. We used verbatim quotes from the participants to validate coding and interpretation of emergent themes. We used an iterative approach to repeatedly review data to allow insights on key themes to emerge [32].

## Results

### Interview participants

In total, we interviewed 16 clinic staff, nine from South Carolina and seven from Texas. Participants were clinic managers or leaders (n = 7), quality improvement directors (n = 3), community health workers (n = 2), or nurses, medical assistants, and other clinical staff (n = 4). Several participants had multiple job roles (e.g. clinic manager and quality director). These participants were counted under the job category that ranked highest (e.g. clinic manager). Of those interviewed, 11 participants took the readiness survey, two did not take the survey themselves but helped with survey distribution among other staff, and three participants did not recall taking the readiness survey but were able to respond to questions based on considering surveys distributed in clinics in general. Participants all worked at FQHCs but represented a range of clinics from small to large and both in rural and urban locations.

### Recruitment and survey distribution strategies may influence survey responses

Interviewees explained their clinics participated in the readiness survey for a number of reasons. Some mentioned it was because they wanted to improve their CRCS rates and thought that the survey would give them a baseline assessment to which they could compare changes. Others mention responding because they wanted the incentive, which they noted as “*extremely helpful*.” Regarding survey distribution among clinics, participants explained that external emails from research teams are not the best way to communicate with clinic staff and noted that staff are more likely to respond to an internally distributed survey from “*an email address that they recognize.*” For example, one participant explained how not knowing the research team who distributed the survey makes it challenging for staff to respond honestly: “*I don't think people want to be totally honest in a situation like this when they're taking a survey and have no idea who the people are they're taking the survey from or where this data is going, or who's going to look at this data.*” However, internal survey distribution also comes with challenges. Participants explained that surveys coming from clinic leadership may make staff hesitant to respond honestly if they are concerned their manager will see the results: “*I just would never want anything to get back to the leadership, saying that I said [something] so the confidentiality would be also a big factor for me.”* For example, one participant explained that because the survey was online and came from a source they did not know (even though the manager told them it was confidential) staff still might question anonymity. This participant suggested that having a conversation (such as an interview or focus group) instead of a survey might make staff feel better. Another staff member suggested putting the message that the survey is confidential in multiple places throughout the survey. She described that they did this for another staff survey and found a larger variety of responses.

### Social desirability contributes to high survey scores

Participants also acknowledged that social desirability was likely a contributing factor influencing the positive scores on the survey. For example, one participant explained the tendency for people to answer positively: “*I think that people on surveys, try to give you the answer that you’re looking for… I think people try to be positive.”* A few participants also said that staff might have responded positively because they did not want their clinic to look bad. For example, one person said, “*If I were going to take a survey with this amount of information in it, I would probably, number one, I would never want my clinic, nor my clinicians to look bad at all like I would never want that to happen, so, I would probably score high or moderately high, just because I wouldn't want them to look bad.”*

### Survey format including the length and scale plays a role in how people respond

Overall, interview participants felt the organizational readiness survey was too long and questions were redundant, which may have contributed to the positive skew. Participants explained that survey respondents could have gotten tired towards the end of the survey and would be more likely to click through so they could finish sooner. Notably, the research team is currently empirically exploring response patterns in a systematic way to understand the validity behind this suggestion. For example, one participant said, “*There were a lot of questions. A lot of them were repetitive. If I remember correctly. You know, it just asks you the same questions, but in a different way.*” Another participant said, “*It seems like sometimes the survey questions were repeated just in a different way.”* Related to this, almost all (94%, *n* = 15/16) participants mentioned that the survey scale had too many options that were hard to differentiate and could cause additional fatigue among respondents. The survey used a 7-point Likert scale ranging from strongly disagree to strongly agree. For example, one person explained “*you either strongly agree or your somewhat agree, what is agree*?”.

### High readiness survey scores may be accurate due to the climate of FQHCs and their efforts towards successful implementation

Some respondents noted that the positive survey responses may reflect the climate of FQHCs and staff passion for improving CRCS rates with patients. Only several participants were surprised at the positive scores; instead, most participants said that the high scores could be accurate and representative. When looking at the scores, one participant said that it made sense for the leadership component in the survey to be high because there is always strong support at her clinic for any screening they implement. However, she suspected that the culture and climate components might be scored higher but, in reality, be lower. Another participant explained how FQHCs are able to pivot and adapt so high scores on a readiness assessment are not surprising: *“health centers are pretty fluid places…. I mean things change all the time. So it might not surprise me that it's higher because I think clinics can be very… dynamic … fluid.”*

Many participants explained that staff who work at FQHCs are passionate about helping their patients. Thus, staff believe that promoting CRCS is good for their patients so they would be inclined to answer positively to survey items, even though implementing the CRCS innovation may end up being more challenging than it seems on the survey. A participant described how FQHCs are founded on team-based care and in order to implement a new CRCS EBI, staff will be on board because they know it is a benefit for the patient. Further, the participant explained that it made sense to her that our developmental readiness scores were skewed positively because “*people that make up FQHCs are… very different from staff at other places i.e. for-profit. Folks who come to work at FQHCs… really believe in the mission and the vision of the health centers. We know that it is to meet the needs of an underserved, under-resourced community…. So it kind of doesn't surprise me that when it comes to culture and climate and even leadership, that those* [readiness scores] *are higher simply because again, if it is in your mission or your passion to serve this particular type of patient population, then you work well in that culture, but you also work very hard to facilitate the culture that exists in community health centers because it is one that you’ve got to work together.*”

Most participants also said that survey responses could vary based on how far along a clinic was in the implementation process. One participant explained that she thought the high scores observed were reflective of being accurate because CRCS is an important part of their clinic: “*I feel like that. That would be accurate.* Another participant explained that CRCS has been a “*stable process,*” is “*routine*” at her clinic, and “*everyone feels comfortable with it.*” The clinic she works at has fully implemented all four CRCS EBIs (patient reminders, provider reminders, provider assessment and feedback, and reducing structural barriers), so she explained that the high score on the survey could be an accurate reflection of the clinic’s readiness for CRCS: “*I do know, especially here at our facility, that it* [CRCS] *was a big push with all of the leaders…. I think the answers and the survey we're correct, and in the leadership I mean it's also a UDS measure. So I know for us, that's another big thing is we're trying to get our numbers up, and we do care about our patients as well that we were just trying to figure out the best process to do it.”*

### Measuring organizational readiness through individual perspectives is challenging due to the complexities of implementation and job silos

Interviewees discussed how different job roles in the clinic setting might have contributed to staff not understanding the survey purpose or survey items. One participant said that when staff do not have a quality improvement mindset, it is difficult for them to understand the concept of readiness. Although there was an “I don’t know / not applicable” response option on the survey scale, participants noted that staff might be hesitant to say they “don’t know” and would instead just select a positive response (e.g. strongly agree). Participants described how clinic staff might not understand how their position connects to the overall CRCS implementation process. One participant explained that an individual’s job role could impact the level of knowledge and ultimately, their readiness scores: “*given the difference in education levels with providers versus medical assistants versus clinic managers, there may have been something lost in translation*.” Related to this, another participant explained that the length of time someone has worked at the clinic may influence their attitude towards implementing a new EBI. For example, physicians who have been at clinics for over ten years might be more resistant to change. However, in general, participants noted that FQHCs have a lot of staff turnover.

Interviewees expressed that assessing organizational readiness with just one survey is challenging. One participant explained, “*I don't think that a survey is going to accurately capture* [readiness]*.*” She went on to explain that there is a difference between the academic lens and actual practice. In discussions with participants, there remained a gap between the research team’s academic understanding of readiness and what is understood by the clinic managers and staff. Some participants were confused by the overall concept of readiness. Several participants described that assessment should come from a variety of methods, including more direct observation from a ‘hands on’ and/or tailored approach for understanding organizational readiness in the clinic setting. Building on this, participants suggested this format could help educate staff on the concept of readiness and answer any questions in conjunction with data collection. For example, one participant explained, *“Maybe it should be a group project rather than an individual one, because there were parts that not everybody could answer, but it was required of the survey. This, would allow for discussion and clarification of questions.”* These individuals did not believe that the group format would bias answers or reduce participation. Of note, the research team has alternative tools that can be used for group processing, assessment, and intervention; the purpose of this study was to understand the accuracy of the survey specifically.

## Discussion

This study set out to improve our understanding of the readiness survey experience and why a sample of FQHC clinics consistently reported high readiness scores. Our study found numerous potential explanations for high scores, including concerns about confidentiality, social desirability bias, knowledge of respondents about the survey content (given their clinic role), and the survey format. Specific to the survey format, we found that survey length and response scaling may have impacted scores and the overall survey experience. A more detailed and systematic analysis of response patterns is currently underway with the research team.

One highly relevant finding was the participant’s concerns about survey confidentiality and the risk of negatively portraying their clinic, which may have led respondents to score their clinics in a favorable way. The importance of survey confidentiality is well-documented in the literature– a standard survey recommendation is to explain how responses will remain confidential [33, 34]. This recommendation is consistent with our findings, which indicated that messages provided throughout the survey could be a helpful way to remind respondents how their data will be protected. This finding also highlights the importance of survey distribution and initial messaging for obtaining accurate ratings. Participants suggested the need for psychological safety in survey design, and factors such as who distributes the survey and the reasons for taking the survey play a role in participant’s perceptions of how safe it is for them to rate their clinic accurately.

Related, the specific issue of social desirability emerged as a potential contributor to the high scores across the sample. Social desirability is a common issue with self-reported surveys [35], particularly when asking about sensitive information [10, 36]. Our findings provide further evidence that social desirability can be an issue with surveys. In this case, the sensitive information was related to making the clinic look bad to outsiders and/or pressure to present their clinic in a positive manner. In other utilizations of the survey (outside this study), the research team explains that the results will be used for formative evaluation and clinic improvement. This tends to increase variability in responses, though an empirical exploration of the impact is required. Interestingly, during our interviews in this study, many participants spoke about the high general capacities of their clinic – e.g., strong culture/climate, high staff capacities, good leadership. This general liking of their clinic may have contributed to the desire to present the clinic in a positive manner, even though dimensions other than general capacities were being rated. In other words, it appears that their *likingness* of their clinic increased ratings. Further empirical exploration of this phenomenon is required.

Our findings also indicated that respondent roles may be a contributor to higher scores. This is because some clinic employees may be less involved in the implementation process, and thus may not know how to respond to questions about implementation readiness, leading them to agree with most questions. In previous qualitative work in FQHCs, there was a reported comprehension gap in understanding readiness subcomponents when interviewing medical assistants compared to quality improvement staff [37]. Our findings further suggest some positions within a clinic may be more knowledgeable about certain implementation constructs. Therefore, it is important to recruit participants that can best respond to survey questions as previous studies indicate [38]. For example, clinic managers likely have a deeper understanding of the clinic’s general capacity. Using a targeted recruitment strategy is ideal for obtaining valuable and informative information.

Researchers have widely cited issues with survey fatigue in implementation studies given the numerous constructs often assessed [39–45]. Survey fatigue can lead to ‘straight-line answering’ (answering the same for several multiple-choice questions), which lowers the overall quality of the survey data collected [42]. This straight-lining phenomenon resulting from survey fatigue may be another key contributor to skewed distributions observed in the sample of FQHCs. Respondents in this study indicated that the survey length and response scale (a 7-point scale) could have contributed to survey fatigue. Potential motivational messaging throughout the survey may help to encourage respondents to maintain focus and complete surveys. Prompting respondents who completed survey items very quickly with an automated note (e.g. this was likely too fast to respond accurately, would you like to reconsider your answers?) has been shown to reduce satisficing and achieve more accurate responses [46]. Additionally, providing feedback when a respondent fails an attention check question can lead to increase in measurement quality [47]. This issue is being further explored by the research team, as preliminary analyses suggest the issue is a bit more complex than simple ‘straight-line answering’.

Lastly, respondents also reported that high scores may be accurate because participating clinics were in fact doing well across the readiness components and subcomponents. Previous studies in the FQHC setting have reported high scores among similar constructs. For example, a study examining organizational culture of FQHCs implementing behavioral health programs reported high scores on several cultural domains (e.g., training and meeting patients’ needs) [48]. This finding is consistent with our study, as many FQHC staff members explained that they are passionate about helping their patients.

Our findings indicate there are potentially multiple contributors to high scores, which have several implications for the readiness survey and other implementation studies using self-report surveys. First, to address confidentiality concerns, researchers need to establish strong partnerships with their collaborators and build trust among survey respondents [49]. Researchers also need to provide explanations of how survey data will be used and reported back to organizational leaders. Further, messaging should be included on surveys to communicate data sharing and protection procedures. Second, given the issues with social desirability bias, researchers may include social desirability questions (or scales) to better assess the potential impact. This is especially important during survey development and validation efforts.

An additional lesson learned from this study was that participant recruitment matters greatly in organizational implementation research. Based on organizational role, different staff members may have different levels of understanding of the innovation. There is a balance between wanting multiple, diverse perspectives and asking people for information they do not know enough about to accurately respond. Researchers are encouraged to engage organizational partners to help provide guidance about which roles may be able to speak to certain topic areas, and including skip patterns in survey design to facilitate more accurate responding. For example, quality improvement officers may be the most informed about specific implementation processes in the clinic, whereas all clinic staff may be able to speak to the overall culture. Thus, researchers should work with partners to identify those who can best answer questions on readiness concepts and implementation outcomes. Additionally, it is important to collect information from respondents about their knowledge of the topic areas, their confidence answering survey questions about certain topics, and the amount of time they have worked at their organization. This information can be used to obtain a better sense of who is contributing data in the sample of respondents.

The study has several strengths including this is one of the first we know of to explore higher scores in implementation survey data. Second, it uses follow-up qualitative data to understand quantitative data collected. Results from this study will be used to inform changes in the next iteration of survey data collection. For example, we have included questions related to social desirability into our survey. Limitations of our study included the length of time between survey data collection and follow up interviews made it challenging for some participants to remember the survey as they reflected on the interview questions. Additionally, in discussions with participants, there remained a gap between the research team’s academic understanding of readiness and what is understood by the clinic managers and staff. There appeared to be confusion over what was meant by readiness by participants. Furthermore, interviews were done by two members of the study team who do not have experience working directly in clinics, but being a member of the research team may have influenced the participant-researcher interactions. Finally, managers indirectly reported varying approaches to management of the CRCS intervention and their staff, and it is not certain how this impacts staff understanding of readiness.

In conclusion, the findings informed several lessons learned for future measurement work conducted in the clinic settings. First, research teams should be intentional with survey distribution and messaging around the survey purpose to avoid concerns with confidentiality and address social desirability. Second, overall survey length and survey scales should be concise to avoid response burnout. Third, clinics may benefit from tailored and ‘hands on’ approaches in measurement of implementation and organizational readiness. Fourth, different job roles and experience in the clinic can impact staffs’ understanding of the overall processes and therefore, impact survey responses.

## Conclusions

Overall, many factors including survey length, social desirability, confidentiality, and survey settings can influence survey scores. Being aware of these factors may help researchers incorporate ways to mitigate survey response biases. Lessons learned from perspectives of survey respondents in our study can be applied to other measurement work and surveys conducted across public health research. There are multiple opportunities for future work to expand on our findings. In particular, examining ways to assess social desirability in implementation research, empirically testing the benefits of building partner trust, and designing ways to simplify the survey format and include motivational messaging. Self-report surveys are an important approach to gaining valuable data about implementation. Therefore, we need to continue to improve on our data collection and measurement for advancing implementation research and practice.

## Supplementary Information

Supplementary Material 1.

## Data Availability

The interview data generated and analyzed during the current study are not publicly available to protect the privacy of those interviewed. The interview guide was developed specifically for this study. The full interview guide is available as a supplementary file.
