# Supplementary material for: Organizational readiness for implementation: a qualitative assessment to explain survey responses
Source: BMC Health Serv Res. 2025 Jan 7;25:36. doi: 10.1186/s12913-024-12149-8 (PMC11705772; doi:10.1186/s12913-024-12149-8)
Supplement: Supplementary file 1 — Supplementary Material 1. [file 12913_2024_12149_MOESM1_ESM.pdf]

Introduction:

Thank you for agreeing to talk with me today to discuss the results of the readiness survey your health center participated in. As a reminder, the overall goal of our project is to improve our survey. We've just finished our first phase of the project, where we had a large number of clinics take our survey. When we looked at the results, we expected to see both low and high scores, but most clinics rated readiness for implementing CRCS evidence-based approaches very highly. So now, we are trying to figure out why this might be. As part of the process of analyzing the data we thought it would be important to hear from participants to better understand the results. Now, I know it has been a while since you have taken the survey, so we will remind you about it. Please answer questions to the best of your ability, and remember there are no right or wrong answers, we just want to make the survey the best it can be and to capture what is actually happening at clinics related to readiness. If you have a hard time thinking back, please just share what you can recall.

Part 1 – Informed Consent
INTERVIEWER:

Do you have any questions before we get started?

The meeting was set to automatically record when we started but I want to make sure that is okay with you? The recording will only be used to help me with my notes.

Added questions esp if they don't remember the survey

Part 2 – Interview Questions
INTERVIEWER:
I want to reiterate that your answers today will not be associated with your name, so I encourage you to be open and honest.

1. To get started, please tell me a little bit on how you first heard about the readiness survey.

2. How often do you or staff get surveys?

3. What are staff's general attitude towards surveys?

4. What does a good survey look like in your opinion?

5. What was your general impression of the survey?
   Probe: Length, response items, questions easy to understand?

6. How clear was the information you received, including the survey purpose and directions?

   Probe: Why you were selected/identified to take the survey? Who in your organization might be better/or best able to answer the survey, and why?
   Probe: What information would be helpful for people to know prior to completing the survey?

Next, I want to share a visual with you. Please take a look at this chart; it shows readiness ratings for Leadership, Culture, and Climate averaged across all the participating clinics.

When the research team looked at the data, we saw that most of the clinics rated their readiness very highly and there were not many clinics who rated their readiness on the lower end. This could be due to the clinics we surveyed really having high scores, but we also wonder if there is something else going on with our survey.

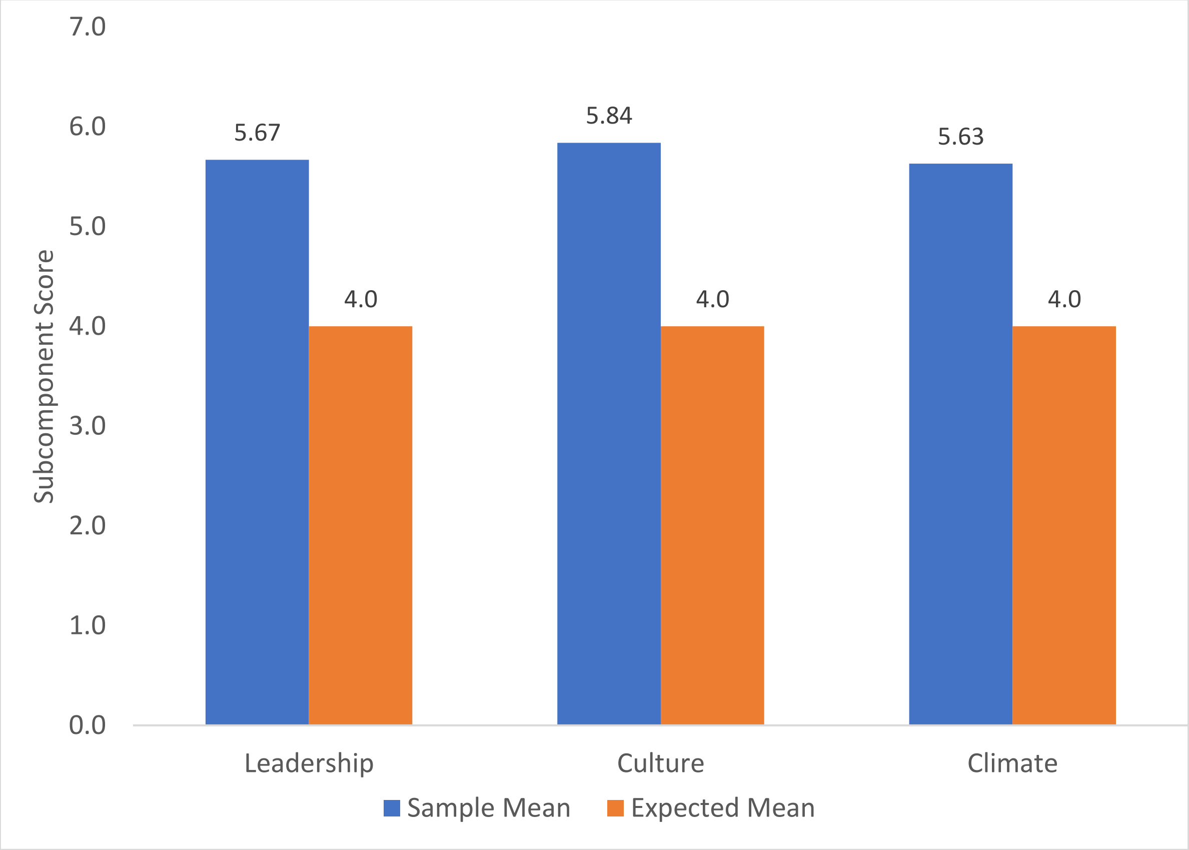

7. Why do you think the ratings look the way they do?

(All of the participants explanations should be followed up on to get as much detail as possible.)

Thank you, your observations are very insightful.

The next questions are going to ask you to remember back to when you took the survey. I know it could be a little hard to remember, but just tell me what you can.

8. We've wondered if the way we sent out the survey impacted the responses we got back. In a lot of instances, we worked with clinic managers to send out the survey to clinic staff. How, if it all, might this process have impacted the way people answered the survey?
   Probe: Concerns about confidentiality of your answers?
   Probe: Concerns about their clinic looking bad?

9. How might your responses to the survey have been impacted by where your clinics was in the process of implementing the CRCS program you were surveyed on: 1) patient reminders, 2) provider reminders, 3) provider assessment & feedback, or 4) reducing structural barriers? For example, if you had already started implementing a new CRCS program versus haven't started implementing it yet.

Scaling/Ratings:
Our team also wonders if our survey's response options need to be revised. As a refresher, we asked people to respond on the following scale [interviewer to show on screen, if possible]:

**Strongly Disagree** (1)	**Disagree** (2)	**Somewhat Disagree** (3)	**Neither Agree nor Disagree** (4)	**Somewhat Agree** (5)	**Agree** (6)	**Strongly Agree** (7)	**Don't Know / Not Applicable**
✓	✓	✓	✓	✓	✓	✓	✓

10. How might these response options have contributed to most of the scores being in the high/agree range (e.g., 5s, 6s, 7s)?
    Probe: What suggestions do you have to improve our response options?

Lastly, I'd like to ask you a few questions about how we could use this information to help clinics build readiness for implementing CRCS EBIs.

11. Our survey gathers a lot of information that we synthesize into a readiness report for each clinic. What types of help might you need in order to address some of the issues that came up in your readiness report?
    Probe: How could we help you in determining what results to focus on improving?

12. Once your clinic has identified those priority results, what else could a team like ours do to help or prepare you to implement evidence-based strategies?
    Possible probes:

a. How would you get started?
b. Who would be involved, and how?
c. What resources would you need?
d. What challenges would you face?
e. What would make the process easier for you?

13. Is there anything else you'd like to share with me that we haven't had a chance to talk about today?

14. Address for gift card?

Thank you so much for taking the time to share your thoughts with me today. I have enjoyed hearing your perspectives.

As a thank you for your time, we will be providing you with a $50 gift card. I will notify the research team that your interview has been completed and they will contact you to arrange the mailing of your gift card.
